# The Integrative Human Microbiome Project

**DOI:** 10.1038/s41586-019-1238-8

**Published:** 2019-05-29

**Authors:** The Integrative HMP (iHMP) Research Network Consortium, Lita M. Proctor, Heather H. Creasy, Jennifer M. Fettweis, Jason Lloyd-Price, Anup Mahurkar, Wenyu Zhou, Gregory A. Buck, Michael P. Snyder, Jerome F. Strauss, George M. Weinstock, Owen White, Curtis Huttenhower

**Affiliations:** 1 National Human Genome Research Institute, National Institutes of Health, Bethesda, MD USA; 2 Institute for Genome Sciences, University of Maryland School of Medicine, Baltimore, MD USA; 3 Department of Microbiology and Immunology, School of Medicine, Virginia Commonwealth University, Richmond, VA USA; 4 Department of Obstetrics and Gynecology, School of Medicine, Virginia Commonwealth University, Richmond, VA USA; 5 Center for Microbiome Engineering and Data Analysis, Virginia Commonwealth University, Richmond, VA USA; 6 Department of Biostatistics, Harvard T.H. Chan School of Public Health, Boston, MA USA; 7 Broad Institute of MIT and Harvard, Cambridge, MA USA; 8 Department of Genetics, Stanford University School of Medicine, Stanford, CA USA; 9 Department of Computer Science, College of Engineering, Virginia Commonwealth University, Richmond, VA USA; 10 Stanford Center for Genomics and Personalized Medicine, Stanford, CA USA; 11 Stanford Diabetes Research Center, Stanford, CA USA; 12 The Jackson Laboratory for Genomic Medicine, Farmington, CT USA; 13 Department of Immunology and Infectious Diseases, Harvard T.H. Chan School of Public Health, Boston, MA USA

**Keywords:** Microbiome, Inflammatory bowel disease, Type 2 diabetes, Reproductive disorders

## Abstract

The NIH Human Microbiome Project (HMP) has been carried out over ten years and two phases to provide resources, methods, and discoveries that link interactions between humans and their microbiomes to health-related outcomes. The recently completed second phase, the Integrative Human Microbiome Project, comprised studies of dynamic changes in the microbiome and host under three conditions: pregnancy and preterm birth; inflammatory bowel diseases; and stressors that affect individuals with prediabetes. The associated research begins to elucidate mechanisms of host–microbiome interactions under these conditions, provides unique data resources (at the HMP Data Coordination Center), and represents a paradigm for future multi-omic studies of the human microbiome.

## Abstract (web summary)

Over ten years, the Human Microbiome Project has provided resources for studying the microbiome and its relationship to disease; this Perspective summarizes the key achievements and findings of the project and its relationship to the broader field.

## Main

Although the ’omics era has accelerated all aspects of biological research, its effects have been particularly apparent in studies of microbial communities and the human microbiome. In the 18 years since the publication of the first human genome, studies of the microbiome have grown from culture-based surveys of the oral cavity and gut to molecular profiles of microbial biochemistry in all ecological niches of the human body^1–3^. Epidemiology and model systems have been used to identify associations between changes in the microbiome and conditions ranging from autism^4^ to cancer^5–7^, and microbial and immunological mechanisms have been identified that affect, for example, the efficacy of drugs used to treat cardiac conditions^8^ or survival during graft-versus-host disease^9^.

Contemporary studies of the human microbiome have also been a source of basic biological and translational surprises, exposing a compelling range of novel findings and open questions. Every human being appears to carry their own, largely individual, suite of microbial strains^10,11^, which are acquired early in life^12–14^, differ between environments and populations^15,16^, and can persist for years^17^ or undergo relatively rapid transitions^18^. Microbial diversity manifests differently in different ecological niches of the body; for example, greater diversity is generally expected in the gut, but can be associated with dysbiotic states and risk of adverse events in the female reproductive tract. The microbiome can be perturbed by conditions such as inflammatory bowel disease and diabetes, but a variety of microbiome-linked health states, and the underpinnings of these links, remain unexplored. How dynamic is the microbiome during processes such as pregnancy or viral infection? Which changes in the microbiome represent causes rather than effects of changes in health? Which molecular elements of a personalized microbiome might be responsible for health outcomes, and how do they integrate with and maintain physiological processes such as the immune system and metabolism? And what ecological elements dictate the success of a microbiota transplant, and why are they successful in treating some individuals and conditions, but not others?

**Fig. 1:**
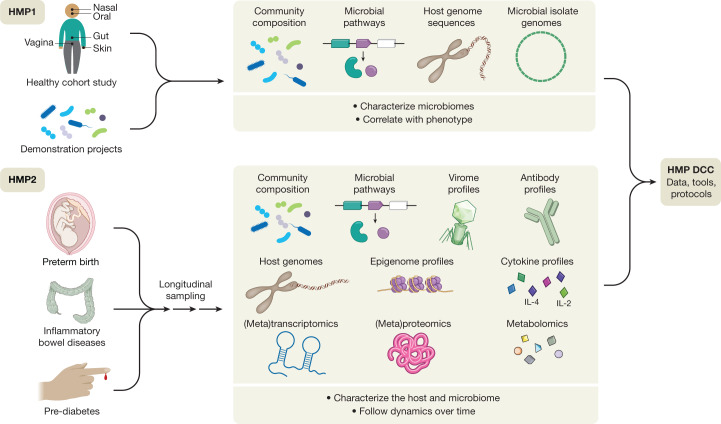
The first and second phases of the NIH Human Microbiome Project. The ten-year NIH Human Microbiome Project (HMP) program, organized into two phases (HMP1 and HMP2), developed reference sequences, multi-omic data sets, computational and statistical tools, and analytical and clinical protocols as resources for the broader research community. The HMP1 focused on the characterization of microbial communities from numerous body sites (oral, nasal, vaginal, gut, and skin) in a baseline study of healthy adult subjects, and included a set of demonstration projects that focused on specific diseases or disorders. The HMP2 expanded the repertoire of biological properties analysed for both host and microbiome in three longitudinal cohort studies of representative microbiome-associated conditions: pregnancy and preterm birth (vaginal microbiomes of pregnant women), inflammatory bowel diseases (gut microbiome) and prediabetes (gut and nasal microbiomes). These studies followed the dynamics of these conditions through multi-omic analyses of multiple measurement types over time, including changes in microbial community composition, viromics, metabolomic profiles, gene expression and protein profiles from both host and microbiome, and host-specific properties such as genetic, epigenomic, antibody, and cytokine profiles, along with other study-specific features. All sequences and multi-omic data, clinical information, and tools from both HMP1 and HMP2 are housed in the HMP Data Coordination Center (DCC) or referenced public or controlled-access repositories to serve as a central resource for the research community.

The National Institutes of Health Human Microbiome Project was one of the first large-scale initiatives to address a subset of these questions^19^ (Fig. 1). Launched in 2007^20^, the first phase of the program sought to determine whether there were common elements to ‘healthy’ microbiomes, in the absence of overt disease. Studies of both a baseline adult population^21–23^ and ‘demonstration’ populations with specific disease states established typical ranges (for some populations) of microbial membership and enzymatic repertoires across the body, combinations of metabolic functions that were either prevalent or strain-specific, and some of the host factors (such as race or ethnicity) that determine this variation. Studies of targeted populations identified ecological states of niches such as the vagina^24,25^, skin^26–28^, and gut^29–33^, among many others (https://www.hmpdacc.org/health/projectdemos.php). This first phase of the HMP (HMP1) thus yielded a wealth of community resources: nucleotide sequences of microorganisms and communities from a large number of isolates, individuals, and populations (http://hmpdacc.org)^34–37^; protocols to support reproducible body-wide microbiome sampling and data generation^38–40^; and computational methods for microbiome analysis and epidemiology^41–47^.

One of the main findings of the HMP1 was that the taxonomic composition of the microbiome alone was often not a good correlate with host phenotype—this tended to be better predicted by prevalent microbial molecular function or personalized strain-specific makeup^21^. This finding served as the foundation for the development of the second phase of the HMP, the Integrative HMP (iHMP or HMP2)^48^, which was designed to explore host–microbiome interplay, including immunity, metabolism, and dynamic molecular activity, to gain a more holistic view of host–microbe interactions over time. This multi-omic program sought to expand the resource base available to the microbiome research community, to begin to address the relationship between host and microbiome mechanistically, and to address the questions introduced above. Disease-targeted projects within the HMP2 were therefore encouraged to use multiple complementary approaches in order to assess the mechanisms of human and microbial activity longitudinally and to provide protocols, data, and biospecimens for future work. These projects included three studies that followed the dynamics of human health and disease during conditions with known microbiome interactions, thus addressing important health outcomes directly while also serving as models of ‘typical’ microbiome-associated conditions of broad interest to the research community. These comprised pregnancy and preterm birth (PTB); inflammatory bowel diseases (IBD); and stressors that affect individuals with prediabetes. These studies, which have now reached the first stage of completion^49–51^, together provide a wealth of information and insights about not only microbial dynamics, but also associated human host responses and microbial inter-relationships. A collection of more than 20 manuscripts to date describe some of these results at https://www.nature.com/collections/fiabfcjbfj, and together they provide a rich multi-omic data resource to be mined by future work (http://www.ihmpdcc.org).

## The vaginal microbiome, pregnancy and preterm birth

Preterm birth can have devastating consequences for newborn babies, including death and long-term disability. In the United States, approximately 10% of births are premature^52^, and the incidence is even greater in lower-resource countries. Environmental factors, including the microbiome of the female reproductive tract, are important contributors to prematurity. Notably, these factors have a greater effect in women of African ancestry, who also bear the highest burden of PTB^53^. Infant mortality has been reduced in recent decades, but the incidence of PTB has not decreased^54^, and progress in predicting individual risk of PTB has stalled. During pregnancy, the maternal immune system maintains a delicate balance of pro- and anti-inflammatory effectors^55^, and contributors to PTB include breakdown in maternal–fetal tolerance, vascular disorders, stress, cervical insufficiency, premature rupture of the fetal membranes, and intra-amniotic infection^56^. Microbial ascension into the uterus is thought to precipitate PTB by disrupting the maternal immune balance, leading to spontaneous preterm labour, and/or by the release of microbial products (for example, collagenases, proteases or toxins) that compromise the integrity of fetal membranes and lead to premature rupture of the membranes^57^.

**Fig. 2:**
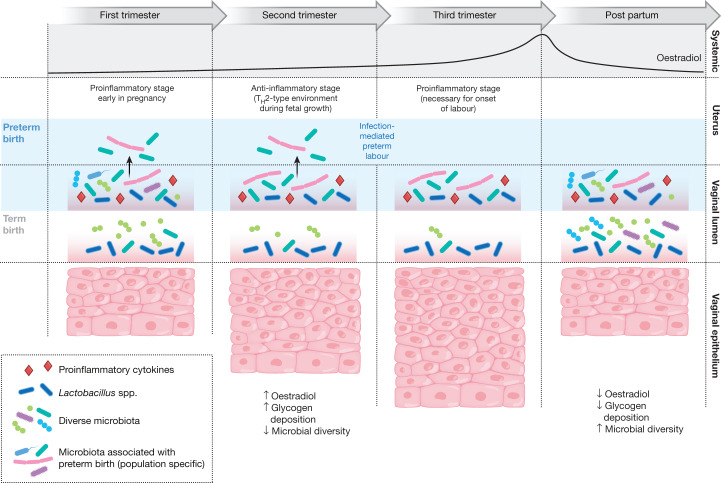
The vaginal microbiome and its relationships with host factors in pregnancy and preterm birth. The MOMS-PI project followed 1,527 pregnancies longitudinally and involved the collection of 206,437 biospecimens for analysis of host and microbial factors (16S amplicon, metagenomic, and metatranscriptomic sequencing; cytokine profiling; metabolomics; proteomics; genomics; and microbial isolate culture). Around 600 pregnancies were analysed in depth to assess features that lead to preterm birth; this analysis identified both host (for example, cytokine) and microbial (for example, ecological and specific strain) factors. As pregnancy progresses, with predictable changes in systemic oestradiol levels, the uterine and vaginal environments undergo various changes. The uterus switches from an early pro-inflammatory condition to an anti-inflammatory condition in the second trimester, and then back to a pro-inflammatory condition before the onset of labour. Meanwhile, specific changes in the microbiome of the vaginal lumen can be associated with preterm birth, possibly through mechanisms involving microorganisms travelling from the vagina to the uterus. The figure depicts an overview of longitudinal changes in the vaginal mucosal ecosystem and uterus during pregnancy.

The Multi-Omic Microbiome Study: Pregnancy Initiative (MOMS-PI) research group, as part of HMP2, characterized the microbiomes of pregnant women to gauge their effects on risk of PTB (Fig. 2). The project followed 1,527 women longitudinally through pregnancy and involved the collection of 206,437 specimens, including maternal vaginal, buccal, rectal, skin and nares swabs, blood, urine, and birth products, as well as infant cord and cord blood, meconium and first stool, buccal, skin and rectal swabs. Subsets of these specimens underwent 16S rRNA gene taxonomic analysis, metagenomic and metatranscriptomic sequencing, cytokine profiling, lipidomics analysis, and bacterial genome analysis. The MOMS-PI team analysed 12,039 samples from 597 pregnancies to investigate the dynamics of the microbiome and its interactions with the host during pregnancy leading to PTB^50^.

These multi-omic investigations identified temporal changes in the vaginal microbiome associated with full-term pregnancies. Women who often began pregnancy with a vaginal microbiome of greater ecological complexity generally converged towards a more homogeneous *Lactobacillus*-dominated microbiome by the second trimester^58^. Interestingly, this trend was most pronounced in women of African ancestry with lower socioeconomic profiles. Although the overall MOMS-PI cohort was demographically diverse, most women who experienced spontaneous PTB at less than 37 weeks of gestation were of African ancestry. The MOMS-PI team (http://vmc.vcu.edu/momspi) also identified signatures of higher risk for PTB in women who experienced spontaneous preterm birth at less than 37 weeks of gestation^50^. Women who went on to experience spontaneous PTB were less likely to exhibit a vaginal microbiota dominated by *Lactobacillus crispatus*, as previously reported in other populations^59–62^, and were more likely to exhibit an increased proportional abundance of several taxa including *Sneathia amnii*, *Prevotella-*related clades, a Lachnospiraceae taxon known as BVAB1, and a *Saccharibacteria* bacterium known as TM7-H1. Notably, these taxa were also associated with low levels of vitamin D^63^, suggesting that the vaginal microbiome might mediate a link between PTB risk and vitamin D deficiency^64^. The signatures of PTB were also reflected in metagenomic and metatranscriptomic measurements, and vaginal pro-inflammatory cytokines (including IL-1β, IL-6, MIP-1β and eotaxin-1) were positively correlated with PTB-associated taxa. Conveniently for future possible interventions, the vaginal microbiomes of mothers who experienced PTB were most distinct from those of control mothers early in pregnancy, and a preliminary model to predict risk of PTB was most sensitive and specific using vaginal microbiome profiles from samples collected before 24 weeks of gestation.

The MOMS-PI research group identified intriguing associations between the vaginal microbiota, host response, and pregnancy outcomes that are consistent with the involvement of microorganisms ascending from the vagina in at least some cases of spontaneous PTB. As an essential next step, the contribution of racial and demographic background to the vaginal microbiome in pregnancy with relation to pregnancy outcomes must be fully explored through harmonized, large-scale studies^50^. It is clear that PTB has a complex aetiology^56^. The relative contributions of fetal and maternal genetics and epigenetics, particularly as related to genetic variation of the innate immune system, should be explored. Harmonized large-scale studies would permit the development of population-specific risk assessment algorithms using vaginal microbiome profiles, features from genetic and prenatal (fetal) genetic screens, biomarkers such as cytokines and metabolites, and key clinical features from classic markers of risk including maternal age, body mass index, pregnancy history (including history of PTB), cervical length, and measures of stress and other environmental exposures. With the addition of new data from the microbiome, other environmental factors, and multi-omic inputs, new algorithms promise to improve our ability to predict risk of PTB early in pregnancy, to facilitate clinical trials by identifying high-risk patients, and ultimately to stratify patient populations into treatment groups.

## The gut microbiome and inflammatory bowel disease

Studies of the gut microbiome in gastrointestinal disease have a particularly long and detailed history, especially in complex chronic conditions such as the inflammatory bowel diseases (IBD). IBD, including Crohn’s disease and ulcerative colitis, affects millions of individuals worldwide, with increasing incidence over the past 50 years or more coinciding with multiple factors such as westernization, urbanization, shifts in dietary patterns, antimicrobial exposure, and many more that could influence host–microbiome homeostasis^65^. The microbiome has long been implicated in IBD, potentially as a causative or risk factor^66,67^, as an explanation for heterogeneity in treatment response (that is, some individuals respond well to relatively benign aminosalicylates or corticosteroids whereas others still experience severe inflammation even after surgical intervention)^68^, or as a novel point of therapeutic intervention (for example, by transplantation of faecal microbiota^69,70^). Although meta-omic techniques have been used to identify functionally consistent microbial responses that help to explain the gut microbiome’s role as part of a pro-inflammatory feedback loop in the gut during disease^71^, and a few strains of microorganisms have been shown to be IBD-specific^72^, no comprehensive model of specific microbial, molecular, and immune interactions yet exists to explain the disease’s onset and dynamic progression.

**Fig. 3:**
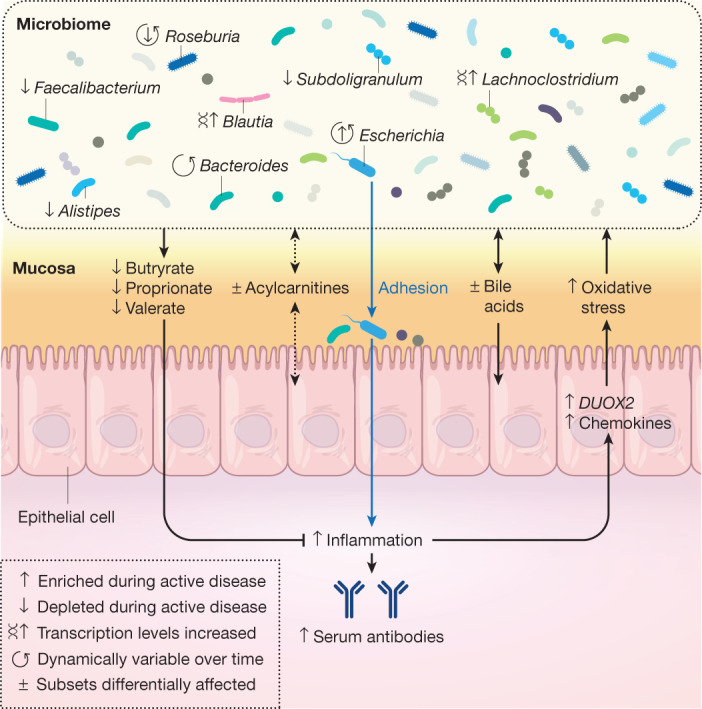
Host–microbiome dynamics in IBD. The IBDMDB followed more than 100 participants with IBD (Crohn’s disease or ulcerative colitis), as well control individuals without IBD, for one year each to assess host and microbial molecular activity during changes in disease activity and gastrointestinal inflammation. Nearly all measured host–microbiome properties showed changes in either activity or stability during disease, including those shown here—not only microbial taxa and microbial transcription, but also host- and microbiota-derived small molecules in the gut, epithelial transcriptional responses at multiple points along the colon, and circulating antibody levels in peripheral blood serology.

Therefore, to better characterize mechanisms of host–microbiome dysregulation during disease, the Inflammatory Bowel Disease Multi’omics Database (IBDMDB) project followed 132 individuals from five clinical centres over the course of one year each as part of HMP2 (Fig. 3). Integrated longitudinal molecular profiles of microbial and host activity were generated by analysing 1,785 stool samples (self-collected and sent by mail every two weeks), 651 intestinal biopsies (collected colonoscopically at baseline), and 529 quarterly blood samples. To the extent possible, multiple molecular profiles were generated from the same sets of samples, including stool metagenomes, metatranscriptomes^73^, metaproteomes, viromes, metabolomes^74,75^, host exomes, epigenomes, transcriptomes, and serological profiles, among others, allowing concurrent changes to be observed in multiple types of host and microbial molecular and clinical activity over time. Protocols and results from the study, further information about its infrastructure, and both raw and processed^76,77^ data products are available through the IBDMDB data portal (http://ibdmdb.org), from the HMP2 Data Coordination Center (DCC; http://ihmpdcc.org), and in the accompanying manuscript^49^.

This unique study design allowed the IBDMDB to identify a variety of differences in the microbiome and host immune response over time during the course of the disease. Indeed, these dynamic changes were of much greater magnitude than were cross-sectional differences among clinical phenotypes, which have been emphasized by previous studies^67,71,78,79^. This was due in part to the prospective nature of the cohort, which recruited patients with Crohn’s disease or ulcerative colitis during both active and quiescent periods of disease, showing that microbial compositions in patients with IBD often revert to more control-like, ‘baseline’ configurations when the disease is not active. By identifying the gut microbial configurations that were most different from baseline—regardless of specific disease state—the study defined a dysbiosis score that called out highly divergent microbial compositions, which share many features common to an overall inflammatory response (for example, tolerance to oxidative stress). This dysbiosis was not unique to the microbial response to inflammation, however, and was associated with other host and biochemical alterations, pointing to new potential directions for management of systemic dysregulation in IBD. These included large shifts in acylcarnitine pools and bile acids, increased serum antibody levels, and alterations in transcription for several microbial species. Concurrent transcriptomics and 16S amplicon mucosal community profiling from biopsies also identified potential host factors that might be able to shape the microbial community, in particular several chemokines, highlighting these as being involved in a potentially dysregulated interaction during periods of disease activity^49^.

The study’s longitudinal multi-omic profiles further allowed researchers to characterize the stability and dynamics of host–microbiome interactions during disease, in particular highlighting ways in which community state and immune responses are distinctly less stable in participants with IBD than in control, healthy individuals. In numerous cases, the microbiome of a participant with IBD changed completely over the course of only weeks (measured as maximal Bray–Curtis dissimilarity to earlier samples from the same subject), whereas such shifts were rare in individuals without IBD. The main microbial contributors to these large-scale shifts from one time point to the next largely mirrored the differences observed in dysbiosis, and the shifts frequently marked the entrance into or exit from periods of dysbiosis. Finally, the study’s long-term, complementary molecular measurements enabled the construction of a network of more than 2,900 significant host and microbial cellular and molecular interactors during IBD, ranging from specific microbial taxa to human transcripts and small molecule metabolites. This network of mechanistic associations identified several key components that are central to the alterations seen in IBD, highlighting octanoyl carnitine, several lipids and short-chain fatty acids, the taxa *Faecalibacterium*, *Subdoligranulum*, *Roseburia*, *Alistipes*, and *Escherichia*, some at both the metagenomic and metatranscriptomic levels, and host regulators of interleukins^49^. Networks of mechanistic associations such as this may provide the key to disentangling the complex system of interactions that results in chronic inflammation in IBD and in other systemic microbiome-linked immune diseases.

## Multi-omics profiling in prediabetes

Type 2 diabetes mellitus (T2D) affects more than 10% of the adult US population, and another 30% show early signs of the disease (referred to as prediabetes)^80^; 70% of the latter will develop diabetes in their lifetime. T2D is characterized by complex host–microbiome interactions^81,82^, but little is known about systemic alterations during prediabetes, their effects on biological processes, or the critical transition to full-blown T2D. Prediabetes and T2D are often associated with insulin resistance, and thus studies of individuals with prediabetes or insulin resistance offer unique opportunities to investigate the earliest stages of diabetes. It is essential to create a global and simultaneous profile of both host and microbial molecules in individuals with prediabetes over time, in order to fully understand the molecular pathways that are affected in people with prediabetes and/or insulin resistance and how these conditions affect both biological responses to environmental challenges (for example, viral infections^83,84^) and the onset of T2D.

**Fig. 4:**
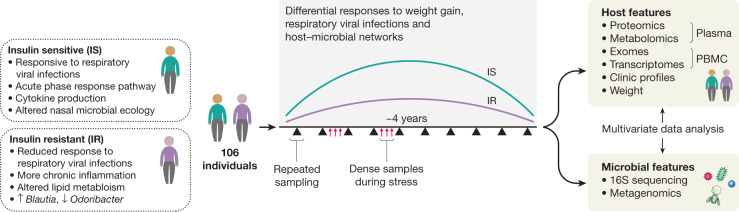
Differential host and microbial responses to dietary perturbations and infectious disease in individuals with prediabetes. For integrated personal ’omics profiling of the microbiome during prediabetes, 106 participants were followed for up to four years, with samples (primarily blood and stool) collected quarterly and additional samples collected during periods of RVI and other stresses. The genomes of the participants were sequenced and measurements of transcriptomes, proteomes, metabolomes, and microbiomes taken at each visit, in addition to clinical details. Insulin-resistant individuals showed differences from insulin-sensitive participants in various measurements, both at baseline and in response to the stresses such as weight loss and RVI.

To better understand T2D at its earliest stages, as part of iHMP, the Integrated Personal ’Omics Project (IPOP)^85^ followed 106 healthy and prediabetic individuals during quarterly periods of health, respiratory viral infection (RVI) and other perturbations over about four years^51^ (Fig. 4). In one such perturbation, a subset of 23 individuals underwent a directed weight gain followed by weight loss^86^. In total, 1,092 collections across all participants were profiled. For each visit, blood was assayed for host molecular ’omics profiling and two types of samples, nasal swabs and faeces, were collected for microbial profiling. Each participant’s exome was sequenced once; otherwise, for each visit, 13,379 transcripts were profiled from peripheral blood mononuclear cells, 722 metabolites and 302 proteins from plasma, and 62 cytokines and growth factors from serum. In addition, thousands of gut and nasal microbial taxa and computationally predicted genes were profiled using 16S rRNA amplicons. All visits were also intensively characterized by 51 clinical laboratory tests. In addition, because of the focus on T2D, a number of glucose dysregulation tests were performed, including measurements of fasting glucose and haemoglobin A1C levels, oral glucose tolerance tests, and tests of insulin resistance.

Baseline measurements were generally stable within individuals, even for long periods of time, with only some analytes changing significantly over time^51^. However, many analytes, such as clinical laboratory measurements, cytokine profiles, and gut microbial taxa (mostly those of low abundance) were highly variable between individuals. Participants who were ultimately insulin-resistant had distinguishable molecular and microbial patterns at baseline from those who were ultimately insulin-sensitive, and an analyte test was devised as part of the study in order to differentiate them. Notably, individuals undergoing RVI or changes in weight showed thousands of specific molecular and microbial changes during these perturbations, and insulin-resistant and insulin-sensitive individuals responded very differently to perturbations. For example, during RVI, insulin-resistant participants showed substantially decreased and delayed inflammatory responses (for example, the acute phase response and IL-1 signalling) and altered gut microbial changes when compared with insulin-sensitive participants (for example, in Lachnospiraceae and Rikenellaceae but not bacilli). Accordingly, there were fewer changes in nasal microbiota in insulin-resistant participants, and both the richness and the diversity of nasal microorganisms decreased during RVI in insulin-sensitive but not insulin-resistant participants. Furthermore, global co-association analyses among the thousands of profiled molecules revealed specific associations in insulin-resistant individuals that differ from those seen in insulin-sensitive participants and vice versa, indicating different patterns of host–microbiome interactions in the two groups^51^.

Another important goal of the study was to assess how host–microbiome multi-omics and related emerging technologies can be used to better manage patients’ health. We found that taking millions of measurements per individual over time enabled the early detection of potential disease states^51,87^. These included early detection of T2D, which developed differently among participants and was better detectable with varied assays; for example, some individuals first exhibited measurements in the diabetic range on tests of fasting glucose, whereas others did so on tests of haemoglobin A1c, oral glucose tolerance tests, or even continuous glucose monitoring. These results, together with detailed characterization of glucose dysregulation over time, illustrate the heterogeneity of T2D development. Overall, the data led to microbially linked, clinically actionable health discoveries in a number of diseases in addition to T2D, including metabolic disease, cardiovascular disease, haematological or oncological conditions, and other areas; these signs were often present before symptom onset, demonstrating the power of using big data, including the microbiome, to better manage human health.

## Resources from the HMP2

**Fig. 5:**
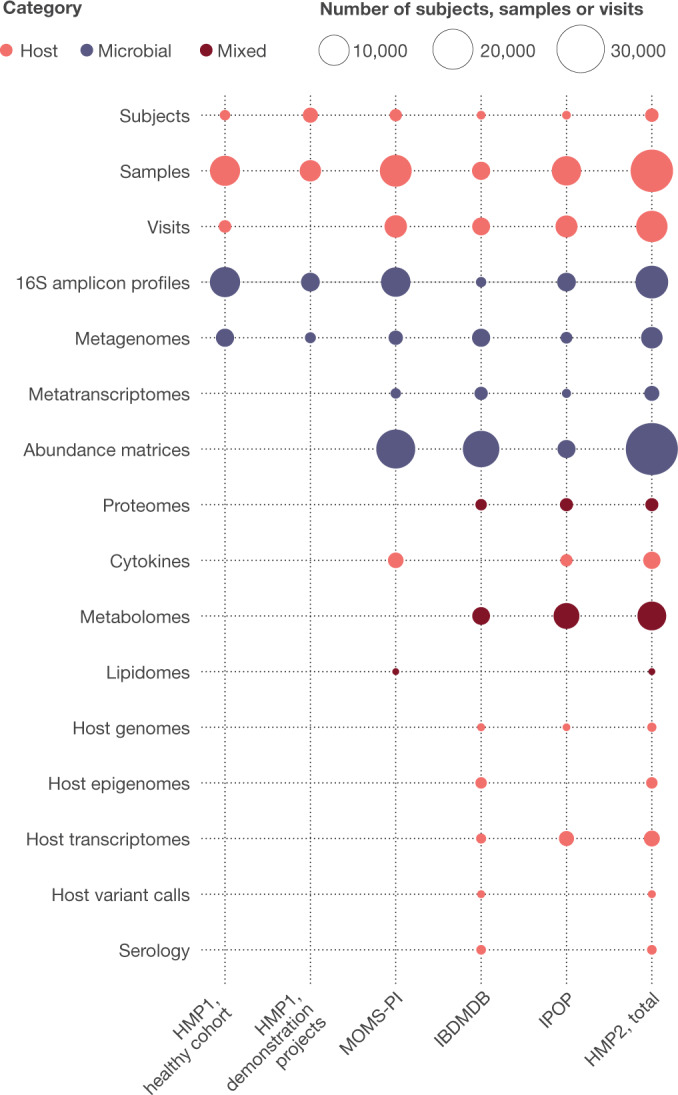
Resources from HMP1 and HMP2 available at the DCC. The HMP DCC (http://ihmpdcc.org) hosts raw and processed data from both phases of the Human Microbiome Project, comprising in total more than 42 Tb of multi-omic data. From the HMP1, these include 16S rRNA gene amplicons and metagenomes from the healthy human subjects (HHS) baseline cohort, as well as resources from demonstration projects (https://www.hmpdacc.org/health/projectdemos.php) and genomes of associated microbial isolates. From the HMP2, these include data that IRBs gave permission to be made publicly available from the pregnancy and preterm birth (MOMS-PI), inflammatory bowel disease (IBDMDB), and prediabetes (IPOP) projects, with links to raw data in other repositories. Additional data deposited elsewhere including microbial reference genomes, HMP1 human genomes, and controlled access data for all HMP2 projects is also linked from the DCC. Categories of data are colour coded, and the number of items in each dataset is indicated by the size of the circles.

Together, the HMP1 and HMP2 phases have produced a total of 42 terabytes of multi-omic data, which are archived and curated by the DCC at at http://ihmpdcc.org and in public and/or controlled-access repositories such as the Sequence Read Archive (SRA; https://www.ncbi.nlm.nih.gov/sra), the Database of Genotypes and Phenotypes (dbGaP; https://www.ncbi.nlm.nih.gov/gap/), Metabolomics Workbench (https://www.metabolomicsworkbench.org/), and others (Fig. 5). All data on the DCC is available for unrestricted use, with a subset of project metadata also being shared when permitted by institutional review boards (IRBs), and other restricted data (for example, human genome sequences and protected metadata) available through controlled access at dbGaP (projects PRJNA398089, PRJNA430481, PRJNA430482, PRJNA326441, phs001719, phs000256, phs001626, phs001523, and others). The formal data models and associated entity relationship schemas produced by all phases of the HMP are freely available at https://github.com/ihmpdcc/osdf-schemas. The DCC website allows users to find, query, search, visualize, and download data from thousands of samples with associated metadata. Once a user has identified a set of files, conditions, subjects, or phenotypes of interest, he or she can add this set to a shopping cart for further operations. Files can then be directly downloaded for use at the user’s local site or in the cloud. The HMP DCC efforts are thus by design consistent with the NIH’s stated goals to make all data generated from NIH funding findable, accessible, interoperable, and reusable^88^. The success of these efforts is evidenced by a consistently high rate of user access to the web resources, with 9,000–12,000 user sessions each month, and a greater throughput anticipated after the publication of these resources here.

## Complementary host–microbiome interactions

Although each of the three HMP2 studies revealed new biology within their respective areas of health and disease, a surprising range of host–microbiome immune and ecological features were common among them. The combination of shotgun metagenomics, untargeted metabolomics, and immunoprofiling was particularly effective, as in all projects this subset of molecular measurements tended to efficiently capture interpretable host and microbial properties that are linked to disease. Conversely, genetic variants were generally difficult to link to the microbiome in such small populations, which were necessary in order to deeply profile multi-omics over time, and we anticipate host sequencing to be more useful when integrated into larger cross-sectional surveys. Another notable property was that, as in most microbiome studies, changes that occurred within individuals, populations, or phenotypes were often much smaller than baseline variation between individuals. This is particularly true at microbiome-relevant time scales, for which repeated measures as rapid as days to weeks were necessary to capture the most specific host–microbiome interactions. Health-associated microbiome interactions can thus manifest in extremely diverse ways among individuals, making a combination of large-scale population surveys with within-subject longitudinal profiles essential for understanding the mechanisms of microbiome-linked disease.

As a result, other aspects of host–microbiome interactions were highly localized and subject-specific within each of the three studies. In all three conditions, microbial changes and associated host responses were strongest when captured at the time the changes occurred, and often within the tissue of origin. It is thus clear from these and other studies that host–microbiome interactions have both localized and systemic effects. Strong local perturbations initiated from either the host or microbial side can induce subsequent spatiotemporal responses that can continue over time and/or in other tissues, presumably with signals carried spatially by circulating small molecules and/or temporally by gene regulation or microbial growth, and involving regulatory circuits with both host and inter-microbial components. Continued coordinated efforts to measure the diverse host and microbial properties involved in each condition will thus be important for developing targeted and, when necessary, personalized therapies for microbiome-associated conditions, as well as for uncovering general principles that govern host–microbiome interactions. Other dynamic interactions that were not measured in all studies, such as an individual’s first microbial exposures near birth and subsequent immune development, may also represent key contributors to baseline microbiome personalization and help to explain disease-linked dynamics based on events that took place years or even decades earlier.

## Next steps in microbiome multi-omics

The collective results of the NIH HMP projects, alongside many other studies, show that the microbiome is an integral component of human biology, with a major role in health and well-being. Inter-individual variability and highly diverse host–microbiome responses over time have driven the development of new methods for population microbiome studies using multiple, complementary longitudinal measurements, as well as highlighting the need to follow such studies up with mechanistic models in order to validate causative associations. The successful close of the HMP program itself has left an enduring legacy of multiple scientific generations of trained human microbiome investigators; provided the resulting community with a wealth of data, analytical, and biospecimen resources; and positioned the NIH and other funding agencies to continue work in a broad range of microbiome-linked conditions^89^. Funding for microbiome science, human and otherwise, is now being coordinated among NIH Centers and Institutes (https://www.niaid.nih.gov/research/trans-nih-microbiome-working-group); other US government agencies including the National Science Foundation, Environmental Protection Agency, Department of Energy, National Institute of Standards and Technology, Department of Agriculture, National Oceanographic and Atmospheric Administration, National Aeronautics and Space Administration, and Department of Defense (https://commonfund.nih.gov/hmp/programhighlights); philanthropic organizations including the Bill and Melinda Gates Foundation, the March of Dimes, the Burroughs Wellcome Fund, the Sloan Foundation, the Keck Foundation, the Juvenile Diabetes Research Foundation, the Crohn’s and Colitis Foundation, and others; and industry and public–private partnerships. Moreover, as complex global projects are launched to tackle aspects of personalized medicine, it is now obvious that it is informative to include components focused on the effect of the human microbiome.

As with any large study, the HMP2 has raised more new questions than it has answered. The aetiologies of baseline inter-individual differences in the microbiome, and of its dynamic changes over time, were not apparent even from the wide range of measurement types incorporated into these three studies and populations. Many immune and biochemical responses appear to be associated with specific strains that are unique to one or a few individual hosts, but it is not clear whether such strains are sufficient or necessary for their associated disease phenotypes. A few mechanisms were identified by which signals in the gut can be transmitted to systemic conditions such as diabetes, but not the specific small molecules or immune cell subsets by which they are likely to be transmitted—particularly in other health conditions that have not yet been studied in such detail. Finally, each HMP2 study was necessarily carried out within a geographically and genetically constrained population, and global differences in early life events, infectious disease exposure, or diet may change how microbiome dynamics contribute to human disease. Human-associated microbiology now clearly extends beyond infectious and gastrointestinal diseases to areas barely imaginable a few decades ago, including metabolism, neoplasia, maternal and child health, and central nervous system function. As the NIH HMP comes to an end, it is clear that its results have revealed a multitude of new avenues of research and technologies for future investigation, and we look forward to new discoveries based on resources from the program and exciting findings yet to come.
